# A comparison of written case notes and the delivery of care in dementia specialist mental health wards

**DOI:** 10.1177/14713012241274994

**Published:** 2024-08-16

**Authors:** Ian Davies-Abbott, Joanne Daunt, Emma Roberts

**Affiliations:** University of Bradford, United Kingdom of Great Britain and Northern Ireland; 8903 Cardiff and Vale University Health Board, University Hospital Llandough, United Kingdom of Great Britain and Northern Ireland

**Keywords:** Case notes, dementia care mapping, language, service development, stigma

## Abstract

**Introduction:** Stigmatising language concerning people living with dementia can cause potentially harmful and dehumanising consequences. Language used about people living with dementia in mental health wards may focus on medical perspectives and suggest custodial relationships with patients rather than person-centred accounts of individuals. This language could have a devastating impact on the provision of person-centred care. This study investigated the relationship between accounts of people living with dementia written in healthcare case notes and clinical practice at three dementia specialist wards in Wales, UK. Language guidance was provided to ward staff to assess whether stigmatising language could be reduced and whether this influenced the provision of person-centred care.

**Methodology:** Dementia Care Mapping was adapted to analyse case note entries for enhancing and detracting accounts of people living with dementia at three data collection points. These were compared to the results of routine DCM observations of care across the three wards. The healthcare case notes of 117 people living with dementia, encompassing 4, 522 entries over ten months were analysed. DCM observations of 38 people living with dementia within the three wards were compared against the case note results. Person-centred language guidance was shared with care staff following each data collection point.

**Results:** Following the provision of person-centered language guidance, the use of personally enhancing language was observed to increase across all three wards. Non-person-centred case note entries predominantly focussed on Labelling language, whilst language concerning Invalidation and Objectification also occurred frequently compared to other DCM domains. Person centred language typically concerned Acknowledgement. A relationship between case note entries and practice was evident in some domains although findings were inconsistent.

**Discussion and Implications:** The findings highlight the importance of addressing stigmatising language in healthcare and suggest that further studies to support the anti-stigma agenda in dementia care are required.

## Background

Stigma is a major barrier to support and advice which promotes an individual’s ability to adjust to their diagnosis and live well with dementia (Alzheimer’s Disease International 2019). The World Health Organisation (2017; 2021) has recognised that stigma is experienced by people living with dementia worldwide and has called for at least one dementia friendly initiative in 50% of all countries by 2025. In Wales, one dementia friendly initiative has identified seven principles for dementia care in Welsh hospitals (Improvement Cymru, 2021, 2022). The Dementia Action Plan for Wales 2018–2022 (Welsh Government, 2018) committed services to promote the dignity, autonomy and rights of people living with dementia and included an action to enhance the use of Dementia Care Mapping (DCM) in care settings.

The stigmatisation of people living with dementia is a social construct, which may be deeply embedded within the language of individuals or larger cultures, potentially including healthcare. This stigma is not exclusive to dementia care with previous literature discussing healthcare providers holding stigmatising attitudes towards people living with mental illness (Subu et al., 2021; Tyerman et al., 2020). Discourse which ignores the personhood and identity of the person living with dementia is potentially dehumanising and challenges the rights of people living with dementia to be regarded as citizens and potentially human beings (Kitwood, 1997; Patterson et al., 2018; Schweda & Jongsma, 2022). Discourse informed by stigma has the potential to lower self-esteem, self-worth and increase feelings of shame and embarrassment (Ballard, 2010; Harper et al., 2019; Mukadam & Livingston, 2012).

Recent anti-stigma programmes utilising on-line education, film, simulated and real contact with people living with dementia have successfully reported a reduction in stigmatising attitudes in the general public (Harris & Caporella, 2018; Kim et al., 2021; Zheng et al., 2016). Whilst the anti-stigma agenda has grown, it is undermined due to having little evidence to support its application (Fletcher, 2019). Although advice around language use is available, the evidence base regarding how to reduce the stigmatisation of people living with dementia is lacking (Herrmann et al., 2018; Kim et al., 2019). Several ‘dementia-friendly’ language guides have been co-produced with people living with dementia (Alzheimer’s Australia, 2015; Care Council for Wales, 2016; DEEP, 2014; Global Brain Health Institute, 2022). However, Kate Swaffer (2014), a person living with dementia has raised concerns that language considered ‘dementia-friendly’ may potentially conceal negative attitudes and behaviour through a superficially acceptable discourse.

Whilst medical records in the UK can be requested through the General Data Protection Regulation (GPDR) (2016), in conjunction with the Data Protection Act, 2018 by individual patients, studies exploring stigmatising language across healthcare case notes are rare (Goddu et al., 2018; Healy, 2023; Park et al., 2021) and have not focussed on language concerning dementia. Healthcare may be assumed to be an area of society which holds fewer stigmatising views, but records describing people living with dementia as ‘challenging’, ‘aggressive’, ‘wandering’ and ‘resistive’ suggest that the person’s behaviour dominates their identity. Without context, these labels present a profoundly negative picture of the person, which will inevitably alter how they are regarded and treated during care interventions. Like Kitwood’s (1997) position regarding the concept of malignant social psychology, healthcare workers do not intentionally use language that stigmatises people in their care but use this language due to long established cultures and a lack of understanding or debate about person-centred case note writing. The purpose of this study was to assess healthcare case notes in dementia specialist wards in Wales, UK for stigmatising language and provide guidance to improve person-centred writing, whilst assessing whether changes in written language impacted on the delivery of person-centred care.

## Methods

The study obtained quality improvement registration with the health board and ethical approval for the involvement of the project lead was obtained through the University.

### Study design

Dementia Care Mapping (DCM) involves structured observation, which seeks to understand the experience of care from the perspective of the person living with dementia (Kitwood, 1997). Indicators of well-being and ill-being in people living with dementia were developed by Kitwood and Bredin (1992) to described observable verbal and non-verbal behaviours. Indicators of well-being include the showing of pleasure, helping others and the initiation of social contact. Indicators of ill-being include physical discomfort, boredom and being left unattended when distressed. During a DCM exercise, a trained mapper observes a person or people living with dementia to understand their quality of life, or well-being, over periods of five-minute time frames.

Mappers also record staff interactions with people living with dementia, which potentially impact on the well-being of the person. Positive interactions are referred to as Personal Enhancers (PEs). Behaviours which are more likely to promote ill-being are referred to as Personal Detractions (PDs). These observations are underpinned by Kitwood’s (1997) psychological model of needs, which includes six interrelated themes with the central theme of Love encompassing an unconditional acceptance of the person, essential to quality dementia care. The bestowment of Love is fulfilled by positive actions within the remaining themes (Comfort, Attachment, Identity, Occupation, and Inclusion). Across these needs, Kitwood (1997) identified 17 PEs and 17 PDs which may be observed during DCM. DCM results are analysed, and feedback is provided to the care staff. The staff are then supported to develop an action plan to improve the provision of person-centred care.

The study applied the DCM observation of PEs and PDs to the accounts of people living with dementia recorded by healthcare workers in clinical case notes across three mental health dementia care wards. These wards provide care for people living with advanced dementia who are typically admitted under the Mental Health Act (Edmans et al., 2022) due to potentially harmful neuropsychiatric symptoms (Wilkins et al., 2019) and whose admissions tend to last months rather than weeks (Adlington et al., 2018; Dementia UK, 2023). Due to the severity of neuropsychiatric symptoms and cognitive impairment experienced by people in these wards, it is often difficult to obtain their perspective of care delivery which makes DCM observations crucial to person-centred service development. The case note analysis was approached identically to the DCM process, with individual writers of case notes left unidentified regardless of their professional status. Following a baseline analysis on each ward, brief language guidance was provided to the ward staff to suggest how PD language could be modified to be person-centred and more informative. The initial guidance was developed using the PE and PD behaviours by the main author and clinicians trained in the methodology. The guidance was revised by the three authors after each data collection point to reflect what had been observed (e.g., many entries regarding abstract ‘confusion’ led to further direction concerning more specific ‘disorientation’). The guidance was provided to the ward staff by the DCM team in the health board during the routine feedback sessions for DCM. The language guidance provided to the wards prior to the final data point is included as supplementary material. DCM was completed by the DCM team within the health board, as part of their routine practice. Both the case note, and clinical practice data collection were informed by DCM User Manual version 8 (University of Bradford, 2005). Concurrently with practice observations and case note analysis, suggestions boxes were fixed at each ward to ask for opinions from anyone accessing the ward regarding the use of person-centred language in case notes. However, despite an explanation of the project and an invitation to share views anonymously, no data was gathered using this process.

### Sample

The study did not contain interventions involving patients, and no data was included that could identify any individual. DCM observations were collected as part of routine practice across the health board and shared anonymously with the project lead. Case notes were analysed as secondary data using the DCM model, initially by the health board mappers and then shared anonymously with the project lead for subsequent analysis. Overall, 4, 522 healthcare case note entries of 117 people living with dementia were analysed over the ten months of the study. The routine DCM practice during this same period included observations of 38 people living with dementia, totalling 1, 824 time frames and representing 152 observed patient hours.

### Data collection and analysis

Both DCM observations and case note analysis were completed every three months across the three wards. The analysed case notes were recorded during the two-week period which aligned to the DCM observations in practice. DCM was completed by trained mappers within the health board who had achieved a higher interrater reliability score than the recommended 80% when the methodology is used for research. Case note analysis using DCM was initially completed by the mappers and then re-analysed by the project lead. Any discrepancies were discussed by the project team to ensure consensus across each case note analysis.

Following baseline case note analysis across the three wards, brief language guidance was shared with the ward staff to suggest how PD entries could be altered to be person-centred. This guidance was reviewed and redrafted before being shared with ward staff again following the second and final data points. A comparative analysis was completed by comparing the occurrence of PEs and PDs in clinical practice and case notes during each two-week period of observation and analysis. Due to the studies experimental use of DCM for case note analysis, a simple comparison method was employed (Bolbakov et al., 2020) using the binary relationships between the PE and PD behaviours observed in the case notes to those observed in clinical practice. This approach was influenced by qualitative comparative analysis as a method for identifying causality in complex systems (Hanckel et al., 2021), for although the occurrence of entries and behaviours could be quantitatively illustrated, the observations and entries were inherently qualitative. The occurrence of PE/PD language and behaviours were compared across the three data points by their percentile increase or decrease. As the first attempt to use DCM for case note analysis, this study acts as a pilot for this methodology and whilst it has been argued that only descriptive statistics should be used in this form of study (Kunselman, 2024), we have trialled some inferential analysis. This descriptive data was used to acquire Spearman’s rank correlation co-efficient (r) scores to measure the dependence between language and behaviour results. A *p*-value was then calculated for each (r) to assess the statistical significance of the correlation analysis.

## Results

### Case note findings

Overall, a reduction in PD language was observed during each data collection period following the provision of language guidance with 59% of all case note entries containing PD entries at baseline reduced to 34% at the second data point and 28% at the final data point. Ward C observed the lowest occurrence of PD language during the second data point, but this was the only ward not to see a continuous improvement over the three data points. The occurrence of PD entries is presented in Table 1.

**Table 1.** Overall occurrence of PD language in written case notes.

	Ward A (%)	Ward B (%)	Ward C (%)
Data collection point 1 (Baseline)	62	64	51
Data collection point 2	49	29	23
Data collection point 3 (Final results)	33	24	24

Although the analysis was completed using the 34 indicators of PD and PE language, only four indicators occurred over 20 times across all three data points. These encompassed 3 PDs (Labelling, Invalidation and Objectification) and 1 PE (Acknowledgement).

Labelling was indicated when case notes described the person as or by a behaviour e.g., the person being described as aggressive, a wanderer, interfering. Other examples indicated an engagement or lack of engagement with interventions e.g., resistive, or non-compliant. Whilst the language guidance did not suggest that particular words could not be used it did indicate a need for context rather than positioning the person as ‘aggressive’ or ‘non-compliant’. The only exception was the entry ‘no management problem’ which does not provide any insight into the person’s experience and therefore should not be used. Labelling was the dominant PD across all three wards at baseline (A: 36%, B: 48%, C:47%) and although occurrences were reduced by the final data point (A: 11%, B: 15%, C: 22%) it remained the dominant PD in wards B and C.

Invalidation was observed in entries which did not recognise the reality of the individual. It was typically observed when the person was described as ‘confused and disorientated’. The language guidance asked staff to consider the person’s disorientation from a strengths-based perspective by considering time, place, and person e.g., whilst a person may not be oriented to time or be aware they are in hospital, they may still recognise family members. Following baseline (A: 20%, B: 8%, C: 2%), Invalidation was the dominant PD in ward A. Whilst ward B and C observed a reduction in this PD at the final data point (B: 5%, C: 1%), ward A observed no change (20%).

Objectification, the description of a person in a non-human manner, typically occurred when interventions were described without an acknowledgement of the person e.g., the person described as being ‘moved’ somewhere without any indication of means, ability, or assistance. Similar entries were observed related to mealtimes and personal hygiene. Although overall less common than the other regularly observed PDs, the language guidance did not appear to significantly impact on occurrences of Objectification from baseline (A: 3%, B: 3%, C: <1%) to final data collection (A: 2%, B: 3%, C: 2%).

Unlike other commonly observed entries, Acknowledgement was indicated in person centred case note writing. This illustrated that staff were recording the likes and dislikes of people on the ward. There was no guidance created for this entry on the language guidance sheet as it was felt that other areas of guidance (e.g., asking for more context) would support this PE and it was preferable to avoid repetition to make the guidance easier to use. Although a higher occurrence of Acknowledgement was observed at the end of the study on two wards (Baseline to final data collection, A: 2%.to 4%, C: 1%–2%), the degree of change was small, and no change was observed in ward B (2%–2%).

### Synthesis with DCM results

During the project, two wards observed an increase in PE practice behaviour from baseline to the final data point across observed PE and PD behaviours during routine DCM observations (A: 61%–80%, B: 66%–71%). There was a moderate positive correlation between the two wards, r = .5; however, the relationship was not significant (*p* = .766). This increase in PE behaviours corresponded with the decrease in PD language across these wards. Whilst ward C did plateau at the final two data points regarding PD language it was the only ward to see a decrease in PE behaviour (60%–53%).

**Figure 1.**
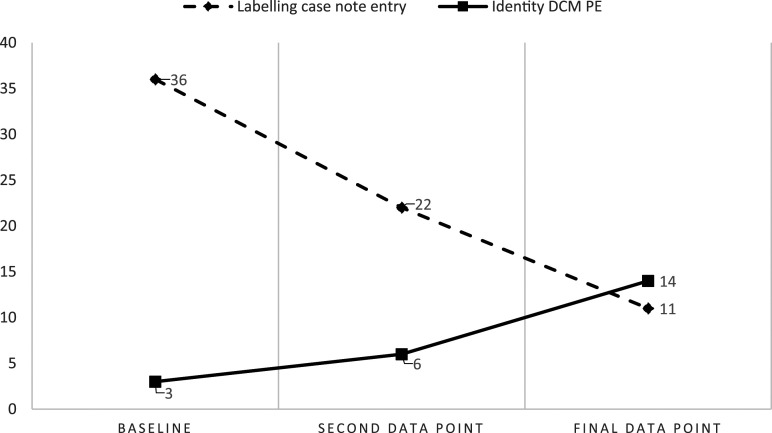
Occurrence of Labelling case note entries compared to enhancing Identity behaviours in practice in ward A.

When practice behaviours and case note results were compared using the themes from Kitwood’s (1997) psychological needs model, the results had clearer affinity in some domains. In ward A, the reduction in PD language concerning Labelling (36%–22% to 11%) was observed concurrently with an increase in PE behaviours within the related psychological need of Identity (3%–6% to 14%). There was a high negative correlation (as intended) between the reduction in Labelling language and heightened PE Identity behaviours, r = −1, although it did not reach significance (*p* = .5). This is illustrated in Figure 1.

Other domains in ward A also observed some symmetry between case note language and practice with the highest incidence of PE behaviour (28%) observed within Occupation alongside an absence of PD language (0%) concerning Objectification at that second data point. Increased PE language relating to Acknowledgement (2%–4%) also corresponded to increased practice PEs concerning Attachment (3%–11%) from baseline to the final data point. However, whilst the baseline and the final data point results suggested a relationship between language and behaviour, the inconsistencies at the second data point (Acknowledgement: 2%, Attachment: 16%) resulted in no correlation or significance (r = 0, *p* = 1). The increase in PE practice behaviour in Attachment was also inconsistent with the case note entries concerning Invalidation, which were unchanged from baseline to the final data point (20%–20%).

**Figure 2.**
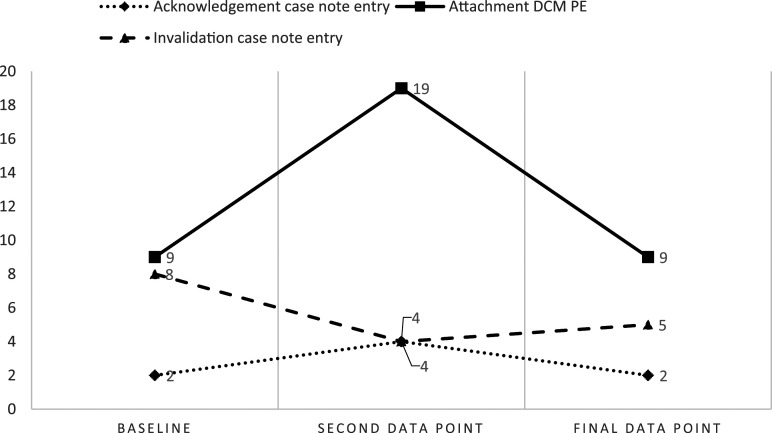
Occurrence of Acknowledgement and Invalidation case note entries compared to enhancing Attachment behaviours in practice in ward B.

In ward B, a relationship between case note writing and practice was observed most closely within the domain of Attachment and the related language PE Acknowledgement and PD Invalidation. As enhancing language (2%–4% to 2%) and practice (9%–19% to 9%) rose and fell, (with perfect positive correlation and significance, r = 1, *p* = 0), the use of detracting language also fell and rose during the same periods (8%–4% to 5%, albeit with strong negative correlation r = .866, but no significance *p* = .333). This relationship is observed in Figure 2.

A similar rise and fall was observed regarding PD Objectification language (3%–1% to 3%) and PE Occupation behaviours (20%–28% to 15%) on ward B. The negative correlation between this malignant language and positive behaviour was very strong (r = −.866) but lacked significance (*p* = .333). A decline in PD Labelling language (48%–15%) from baseline to the final data point corresponded to an overall increase in PE Identity behaviours (9%–15%) although the second data point observed less PE behaviours (9%–4%) despite lower occurrences of PD language from baseline (48%–23%). This indicated moderate negative correlation and no significance (r = −.5, *p* = .765).

There were fewer correlations between observed language and practice on ward C. Improvements in Labelling (47%–22%) did correspond to a small increase in PE Identity practice (7%–8%, r = .5, *p* = .765) and increased observations of PE Acknowledgement in case notes (1%–2%) occurred concurrently with a decrease in PD Invalidation (2%–1%), indicating perfect negative correlation r = −1 and significance, *p* = 0. However, despite the latter positive changes in language, a decrease was also observed in PE behaviours concerning Attachment (10%–5%). Similarly, whilst PE Occupation behaviours increased (3%–8%), PD Objectification language also increased from baseline to the final data point (0.5%–2%, r = .5, *p* = .666).

## Discussion

Whilst the findings were not consistent across all three wards, the correlations between case note language and practice behaviours do support previous hypotheses that non-person-centred discourse leads to potentially harmful consequences, through malignant social psychology, for people living with dementia (Kitwood, 1997; Patterson et al., 2018; Schweda & Jongsma, 2022). Positively, it was also observed that the use of person-centred language can equally lead to better care for people living with dementia on mental health wards. The results of the case note analysis alone illustrate that it is possible to address stigma and add to the previous reports of successful anti-stigma programmes (Harris & Caporella, 2018; Kim et al., 2021; Zheng et al., 2016). Whilst altering language to be superficially ‘dementia friendly’ may conceal negative attitudes and assumptions (Swaffer, 2014), the results do suggest that it is possible to influence more positive viewpoints of dementia in individuals when prompted to consider the ‘dementia friendliness’ of their language, which can lead to real, rather than superficial, practice outcomes.

The results were not consistent across the three wards, with ward A showing no improvement in written language concerning Invalidation, yet consistent with the other wards regarding the reduction in Labelling language. This could be attributed to the case note conventions of the individual wards. Both wards B and C presented a blank page for case note writing, allowing to staff to structure their case note entries without prompts. Ward A, however, used a template for case note writing which asked for a daily review of ‘cognition’, which often resulted in the entry: ‘confused and disorientated’. The language guidance was revised following the baseline audit to support the staff to consider how the entry for ‘cognition’ could provide greater insight into the person’s experience, but this did not impact on ward A’s results within this PD. We would argue that the language guidance does indicate how to remove these incidents of Invalidation from the case notes, even when using a template. However, the template embeds this language even further into case note writing routines on ward A and in these circumstances ward staff may require more than a written guide to question their everyday language.

Both correlation (r) and significance (p) indicators varied between wards and variables. This variance does confuse the clinical implications of the study, as the relatively small increase of Acknowledgement in case notes (1%–2%) occurring alongside a decrease in Invalidation (2%–1%) had a perfect negative correlation (r) score (r = −1) and significance (*p* = 0) on ward C. Comparatively, the reduction in Labelling on ward A (36%–22% to 11%) and the concurrent increase in PE Identity behaviours (3%–6% to 14%) had a similarly perfect correlation (r = 1) but lower significance (*p* = .5). However, the impact of reducing Labelling language from 36% of all case note entries to 11% was clinically more impactful than the 1% changes observed in ward C’s Acknowledgement and Invalidation. Research data contains more meaning than is presented in a *p*-value, which can lead to inappropriate interpretations of results (Andrade, 2019). Less significant *p*-values observed in some of the findings in this study should not indicate to clinicians that detracting language does not have an impact on the person it is used about. This study was the first attempt to analyse the relationship between case note language and practice, and therefore whilst significance statistics may be interesting, studies in their infancy or at their pilot stage should focus on the descriptive results (Lee et al., 2014).

The DCM mappers who shared the language guidance observed that ward staff proactively engaged with the guidance, offering alternatives, and acknowledging that this was a subject they had not previously reflected upon. This was unsurprising as previous studies concerning case note language are few (Goddu et al., 2018; Healy, 2023; Park et al., 2021) and do not concern dementia care. However, this enthusiasm was not replicated in the contributions to the suggestion boxes by either ward staff or ward users.

Whilst co-produced language guides (Alzheimer’s Australia, 2015; Care Council for Wales, 2016; DEEP, 2014; Global Brain Health Institute, 2022) have highlighted the preferred terms used by and for people living with dementia, the guidance in this study was developed using existing standards for quality dementia care through DCM (University of Bradford, 2005). However, the results indicated that not all the 17 PE and 17 PD actions observed in DCM were commonly observed in case note writing.

Labelling language dominated the case note entries at baseline, but the results also evidenced that this was the PD most open to positive change. Whilst the anti-stigma agenda has been hampered due to a lack of evidence (Fletcher, 2019; Herrmann et al., 2018; Kim et al., 2019), this study provides evidence that awareness raising, and the provision of brief guidance can have an impact on the words used about people living with dementia and provides preliminary evidence that this could also result in better person-centred practices. Whilst this was the first attempt to understand the relationship between case note writing and care delivery, the descriptive results indicate that clinicians should be embracing person-centred approaches to writing about people living with dementia. Whilst the impact on care delivery needs further study, the current evidence suggests that person-centred writing causes no harm and may have a positive impact on people living with dementia. The sharing of the brief guidance with care staff is a simple, low-cost intervention which has credible clinical implications.

### Strengths and limitations

The study included a substantial amount of case note entries and hours of practice observation, which supports the validity and transferability of the results. As the data collection was completed by DCM mappers existing already within the service, there were no practice alterations required (e.g., the introduction of DCM), which again aids the transferability of the study outcomes. Whilst other studies have explored stigmatising attitudes in healthcare case notes (Goddu et al., 2018; Healy, 2023; Park et al., 2021), this is the first to explore this subject in the context of dementia and has even greater value due to comparing case note entry results to care delivery.

Whilst identifying the 3 PDs and 1 PE which occurred most frequently is a valuable finding, it would have supported the anti-stigma agenda to complete further analyses using these 4 themes within a redefined context, outside of DCM. This would support non-DCM mappers to consider how they could use this same approach in their own analyses of case notes in the future. By following the DCM guidance, the study did not identify staff members who created case note entries or delivered care. Therefore, it was not possible to consider whether a person’s culture or ethnicity impacted on the results. Similarly, although case note entries were written in English, it was not identified whether English was the first language of all care staff. By developing an analysis outside of the DCM structure, further studies will be able to explore the impact of these demographic details.

Further studies should employ more complex statistical analysis methods. For this study, it was hypothesised that Kitwood’s (1997) psychological model of needs could be used for case note analysis but due to the novel approach, only a simple comparison between the two sets of data was attempted.

The failure of data collection using suggestion boxes does align to the assumption that this is an under discussed practice area and to gain perspectives of people on the wards in future studies using a more focussed data collection method (e.g., focus groups) may be more successful.

The application of correlation and significance statistics to this data was only introduced following the collection of the data. Although high significance was observed at times, this may have been due to the variation in data sizes rather than accurate indications of significance or its absence. Applying this analysis in future studies on this subject should be planned during the pre-data collection period.

## Conclusion

The provision of brief language guidance, without further intervention, leads to more person-centred case note writing. The conventions of case note writing in the care facility may impair the impact of the language guidance. Whilst not all the findings consistently illustrated that the language used in healthcare case notes directly influenced the delivery of care, the study provides strong preliminary outcomes to suggest that this is an important area to address in research and healthcare. More studies are now needed to explore this aspect of the anti-stigma agenda and should focus on simplifying the analysis, investigating varied care settings and considering multi-lingual contexts.

## Supplemental Material

Supplemental Material - A comparison of written case notes and the delivery of care in dementia specialist mental health wards Supplemental Material for A comparison of written case notes and the delivery of care in dementia specialist mental health wards by Ian Davies-Abbott, Joanne Daunt and Emma Roberts in Dementia

## Data Availability

The datasets used and/or analysed during the current study are available from the corresponding author on reasonable request.
