# Supplementary material for: A comparison of written case notes and the delivery of care in dementia specialist mental health wards
Source: Dementia (London). 2024 Aug 16;24(2):310–22. doi: 10.1177/14713012241274994 (PMC11780970; doi:10.1177/14713012241274994)
Supplement: Supplemental Material - A comparison of written case notes and the delivery of care in dementia specialist mental health wards [file sj-pdf-1-dem-10.1177_14713012241274994.pdf]

Language guidance - descriptions of behaviours from DCM User Manual version 8 (University of Bradford, 2005).

Labelling	Why is this wrong?
Describing the person as or by a behaviour.	Labelling creates stigma which threatens the delivery of person-centred care. Labels are the antithesis of person-centred care as they recognise the behaviour but not the person.
**Labelling**	**Acceptance**
Wanderer/Wandering/Wandersome<br>*This suggests that the person's behaviour has no purpose*	The person is walking.<br>Is this an expression of an unmet need? *Has the person been separated from someone/something? Is the person looking for someone/something or lost?*
Compliant/Non-compliant<br>Resistive/ Not resistive<br>*These suggest the person should 'comply' without considering their perspective.*	The person chose not to....<br>The person did not want....<br>Language should not be custodial or suggest that you have authority over the person.<br>Does the person understand what is being offered?
Aggressive<br>Hostile<br>Irritable<br>Shouting<br>Bad tempered<br>Bizarre<br>Stubborn<br>Argumentative<br>Difficult<br>Interfering - *Include what the person is trying to do (e.g. help, engage) and add that this attention was not accepted by the other person involved. Without this context, 'interfering' appears malicious and does not help us understand how we can help the person.*	Consider the behaviour from the perspective of the person.<br>An expression of unmet need. *What are they trying to achieve?*<br>Be descriptive.<br>*What was happening before? What caused the change in how the person was feeling?*<br>*Be specific about what actually happened.*<br>*What support was given?*
No management problems	There is no alternative to this term. It **SHOULD NOT** be used. It suggests an expectation that the person will present as a problem and other than labelling the person as a problem is otherwise meaningless and adds nothing to the care of that person.

Invalidation	Why is this wrong?
Not recognising the reality of the individual.	The ability to stand in the person's shoes and try to see the world as they do is imperative in person centred care. To deny the person's reality is to deny the lived experience of the person with dementia.

Invalidation	Validation
Describing someone as only 'Confused and disorientated'.<br>*What does this tell us about the person? Are confusion and disorientation different?*	Focus on what the person can do and what they have achieved. Think about time, place and person. What are they orientated to?<br>e.g., Disorientated to time and place. Able to recognise wife/family/staff members etc<br>e.g., Disorientated to place and person. Dining area environment supported him to be oriented to lunchtime.<br>e.g., Disorientated to time and person. Able to locate and use the toilet independently.

Objectification	Why is this wrong?
Describing the person in a non-human manner.	This suggests that people with dementia are unequal to others in regard to their human rights.
**Objectification**	**Collaboration**
When the person is referred to in a manner which may be used for an object rather than a person.<br><br>e.g., Person was moved to..., taken to…	How did the person move?<br>*Did they walk with staff to…?*<br>*Assisted in a wheelchair to…?*<br>This makes it clear that the entry is about a person and tells us something about how they move.<br><br>Think about this for any action e.g., eating, washing, dressing etc.

Infantilisation	Why is this wrong?
Describing a person as childlike.<br>Using patronising language.	Describing a person as childlike suggests that the person should be treated as a child.
**Infantilisation**	**Respect**
The person's behaviour is described as unacceptable.<br>The person is described as 'naughty'.	Consider the unmet need. What is the person doing and what are they communicating?
